# Supplementary material for: Mechanisms and targets of Fcγ-receptor mediated immunity to malaria sporozoites
Source: Nat Commun. 2021 Mar 19;12:1742. doi: 10.1038/s41467-021-21998-4 (PMC7979888; doi:10.1038/s41467-021-21998-4)
Supplement: Supplementary file 1 — Supplementary Information [file 41467_2021_21998_MOESM1_ESM.pdf]

SUPPLEMENTARY INFORMATION

Mechanisms and targets of Fcγ-receptor mediated immunity to malaria sporozoites

Gaoqian Feng, Bruce D. Wines, Liriye Kurtovic, Jo-Anne Chan, Philippe Boeuf, Vanessa Mollard, Anton Cozijnsen, Damien R. Drew, Rob J. Center, Daniel L. Marshall, Sandra Chishimba, Geoffrey I. McFadden, Arlene E. Dent, Kiprotich Chelimo, Michelle J. Boyle, James W. Kazura, P. Mark Hogarth, James G. Beeson

SUPPLEMENTARY METHODS

Expression of recombinant proteins antigens

Sequence selection and modification: We generated recombinant proteins based on the 3D7 allele *P. falciparum* CSP (XP_001351122), expressed in the mammalian HEK293F cell line. CSP contains 397 amino acids and can be divided into several domains: The N-terminal signal peptide (amino acids 1-18), the N-terminal domain (amino acids 19-104) that contains a free cysteine residue at position 25, the central repeat region consisting of 4 NVDP and 38 NANP repeats (amino acids 105-272), and the C-terminal domain (amino acids 273-398) that contains a predicted GPI anchor Omega site at Cys-374. In this study, we expressed two different subunits of CSP representing the C-terminal region (CT) and a fusion between the N-terminal and C-terminal regions (N+C; NANP and NVNP repeats absent). The CT construct contains amino acids 273-383 and represents a slightly truncated form of the CT with a disrupted GPI anchor motif. The N+C construct contains a slightly truncated form of the N-terminus (amino acids 26-104; removing the endogenous signal sequence), which is fused to the C-terminal domain (amino acids 273-383) via a short GGGS linker. To mediate secretion of these recombinant proteins into media and purification via nickel resin

chromatography, a signal peptide for Tissue Plasminogen Activator (TPA), followed by a 6-histidine tag was fused to the N-terminus of both proteins. Both protein sequences were then assessed for potential glycosylation sites (http://www.cbs.dtu.dk/services/NetNGlyc/) but none were identified and so no further modifications were made to the protein sequences.

Generation of protein expression vectors: The CT and N+C protein sequences were used to generate DNA sequences that were codon optimized for mammalian expression and then synthesized (GeneArt, Thermo Fisher Scientific). The synthetic genes were supplied in a Puc vector, and then cloned into pcDNA 3.1+ using *Xho*1 and *Bam*H1 restriction sites. The final plasmids were quantified and used to transfect HEK293F cells.

HEK293F cell culture and transfection: HEK293 Freestyle™ cells (Thermo Fisher Scientific) were cultured following the manufacturer's protocols. In brief, cells were cultured in Erlenmeyer shaker flasks (125ml, Corning) with FreeStyle™ 293 Expression Medium (Thermo Fisher Scientific) at 37°C, 8% CO_2 at 135×rpm on an orbital shaker. Cells were counted using the trypan blue (0.4%; Thermo Fisher Scientific) cell exclusion method using *Countess*™ Cell Counting Chamber Slides (Thermo Fisher Scientific) and the Countess™ automated cell counter (Thermo Fisher Scientific). HEK293F cells were transfected for protein expression following the manufacturer's protocol (Thermo Fisher Scientific) with minor alterations. On the day of transfection, cells were centrifuged (700 *g* at 4°C, 10 min) and resuspended in HEK293F expression media with 1:100 antibiotic/anti-mycotic solution (Thermo Fisher Scientific) at a final density of $1x10^6$ cells/ml. For a 30 ml transfection, 90 µl of Polyethylenimine (PEI) transfection reagent (25kDA linear; Polysciences; stock 1 mg/ml) was added to 0.6 ml of OptiPro™ Serum Free Medium (Thermo Fisher Scientific) and incubated for 5 minutes. This solution was then added to the DNA solution (30 µg of purified plasmids and 0.6 ml of OptiPro™ Serum Free Medium (Thermo Fisher Scientific). After incubating for 10 minutes at room temperature, this final

solution was added to the cells, and then returned to the orbital shaker and incubated. The next day, Lupin (1:40 of 20% w/v, Biotech Solabia) and Pluronic acid F-68 (1:100 of 10% w/v) (Thermo Fisher Scientific) were added. The expressed proteins were harvested 6 days post-transfection by centrifuging cells (700×*g*, 10 minutes) to collect supernatant that was then filtered (0.2 µm membrane) and stored at 4°C until purification. Protein expression was tested for using SDS-PAGE gels and western blot.

Protein purification and dialysis: Harvested media containing the expressed proteins was passed over nickel resin columns (Life Technologies, Thermo Fisher Scientific), washed with 20 mM imidazole/PBS (Sigma-Aldrich), and bound proteins were eluted in several 1ml fractions in 500 mM imidazole/PBS. Eluted fractions were tested for the presence of protein by spectroscopy and SDS-PAGE. Fractions containing protein were pooled, filter sterilized, dialyzed into sterile PBS, and adjusted to a concentration of 1 mg/ml via centrifugation using 10,000MW cut-off filters. Protein size and purity was confirmed by SDS-PAGE.

SUPPLEMENTARY FIGURES

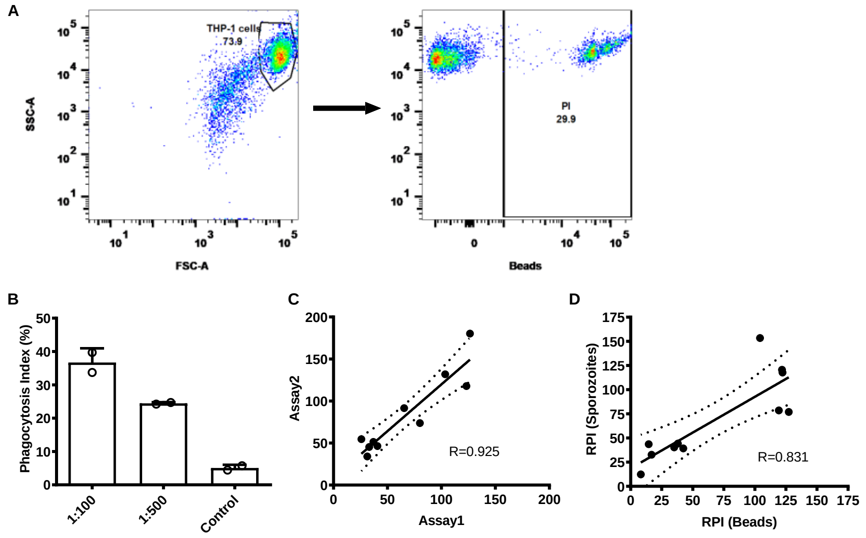

Supplementary Figure 1 Establishement of phagocytosis assay using THP-1 cells.

(A) Example of gating strategy to quantify the level of opsonic phagocytosis by THP-1 cells. This gating strategy was used in Figures 2C-D and Supplementary Figures 1B-D. The THP-1 cell population was gated on size and complexity (FSC-SSC) and the phagocytosis index (PI) was defined as the percentage of THP-1 cells that have phagocytosed fluorescent beads or labelled sporozoites.

(B) Cryo-preserved *P. falciparum* sporozoites were stained with ethidium bromide, opsonized with a pool of Kenyan adult sera and phagocytosed by THP-1 cells. Pooled serum samples from malaria-naïve Melbourne donors were used as negative control. A higher level of phagocytosis was seen when sporozoites were opsonized with Kenyan adult sera. The level of phagocytosis is presented as phagocytosis index which reflects the percentage of THP-1 cells that have taken up fluorescently stained sporozoites (data show mean and standard error from 2 independent experiments).

(C) Reproducibility of the opsonic phagocytosis assay using cryo-preserved *P.falciparum* sporozoites and THP-1 cells (n=10, Spearman's rho=0.925, p<0.001). The sporozoites were opsonized with a selection of serum samples from a Kenyan cohort to represent high, intermediate and low responders based on total IgG to CSP. Phagocytosis assays were performed twice independently. Phagocytosis index was standardized to the percentage of the positive control, which was a pool of Kenyan adult serum. Dots represent the standardized phagocytosis indexes of each individual, the solid line represents the predicted relationship in a linear regression model between the two indenpent assay repeats and dotted lines represent the 95% confidence intervals of the prediction.

(D) Opsonic phagocytosis activity by THP-1 cells was strongly correlated when using CSP-coated beads or cryo-preserved *P. falciparum* sporozoites (n=11, Spearman's rho=0.831, p=0.002). Cryo-preserved sporozoites or CSP coated beads were opsonized with a selection of serum samples from a Kenyan adult cohort to represent high, intermediate and low responders based on total IgG to CSP. The phagocytosis index was standardized to the percentage of the positive control, which was a pool of Kenyan adult serum and defined as relative phagocytosis index (RPI). Dots represent the RPIs of each individual, the solid line represents the predicted relationship in a linear regression model between the RPIs tested using sporozoites and beads, the dotted lines represent the 95% confidence intervals of the prediction.

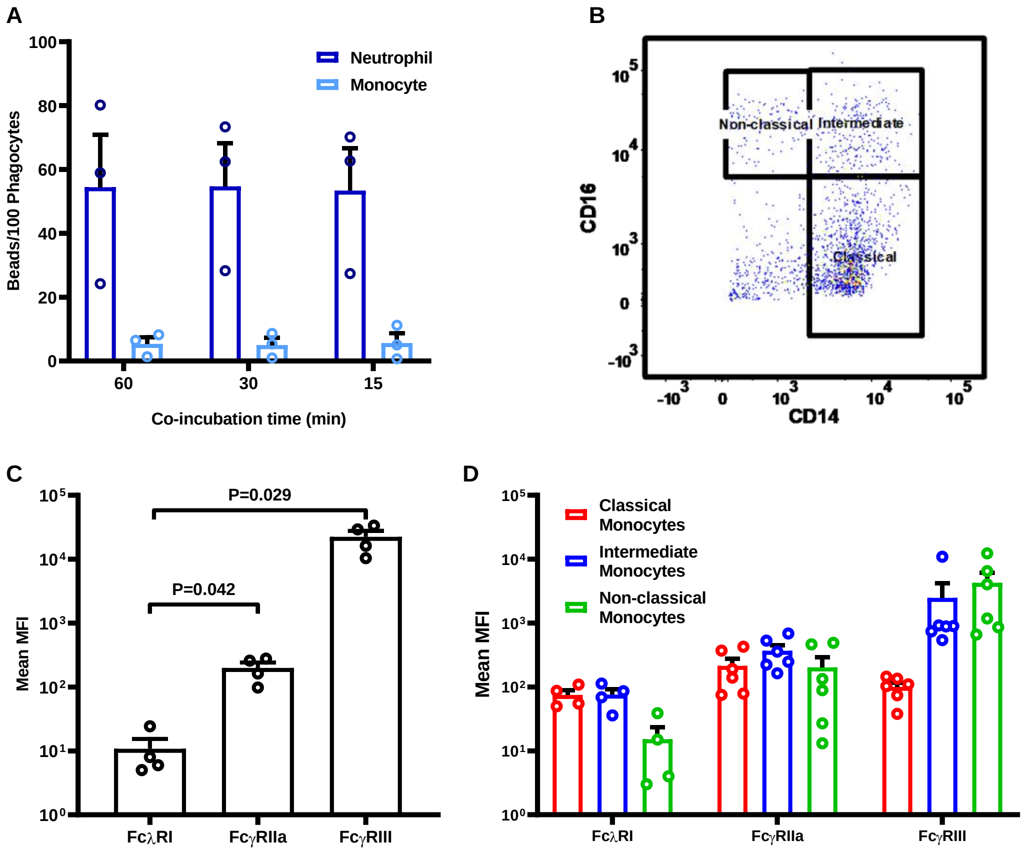

Supplementary Figure 2 Analysis of phagocytic activity and Fcγ receptor expression by monocytes and neutrophils

(A) CSP coated beads were opsonized by a pool of Kenyan adult sera prior to co-incubation with whole leukocytes for variable lengths of time. Neutrophils remained the dominant phagocytes in opsonic uptake of CSP bead at all-time points (Two-way ANOVA P<0.001). Bars and error bars represents the means and standard errors from 3 experiments.

(B) Example of gating strategy for identifying monocyte subsets. This gating strategy was used for Figure 1D and Supplementary Figures 2C-D. The monocyte population was further divided into classical monocytes ($CD14^{high}$ $CD16^{-}$), intermediate monocytes ($CD14^{high}$ $CD16^{+}$) and non-classical monocytes ($CD14^{low}$ $CD16^{+}$) based on surface CD14 and CD16 expression.

(C) Expression of Fcγ receptors on neutrophils was determined by labelling with specific antibodies and analysis by flow cytometry. Bars represent means and standard errors of MFI (shown on a $\log_{10}$ scale) from 4 separate experiments. FcγRI expression on the surface of neutrophils was significantly lower than FcγRIIa and FcγRIII (One-way ANOVA, P=0.042 and P=0.029 respectively).

(**D**) Expression of Fcγ receptors on different monocyte subsets present in whole blood (two-way ANOVA for differences between groups, P=0.035). Bars represent means and standard errors of MFI (shown on a $\log_{10}$ scale) from 6 experiments.

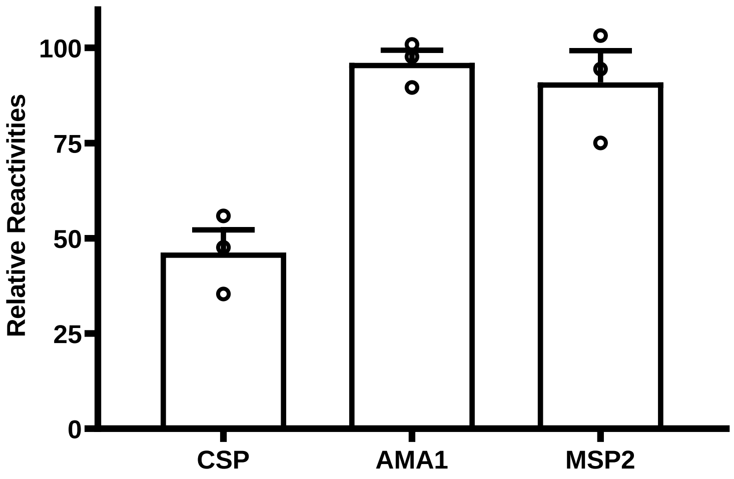

Supplementary Figure 3 Depletion of CSP specific antibodies from a pool of Kenyan adult sera

CSP specific antibodies were depleted from a pool of Kenyan adult sera by incubation with of full-length CSP and NT peptide. The level of total IgG responses against full-length CSP, AMA1 and MSP2 post depletion were expressed as the percentage of IgG responses of the non-depleted sample (relative reactivity). CSP specific antibodies were significantly reduced whereas the IgG responses to AMA1 and MSP2 were unaffected (One-Way ANOVA P=0.002). Bars and error bars represents the mean and standard errors from 3 experiments.

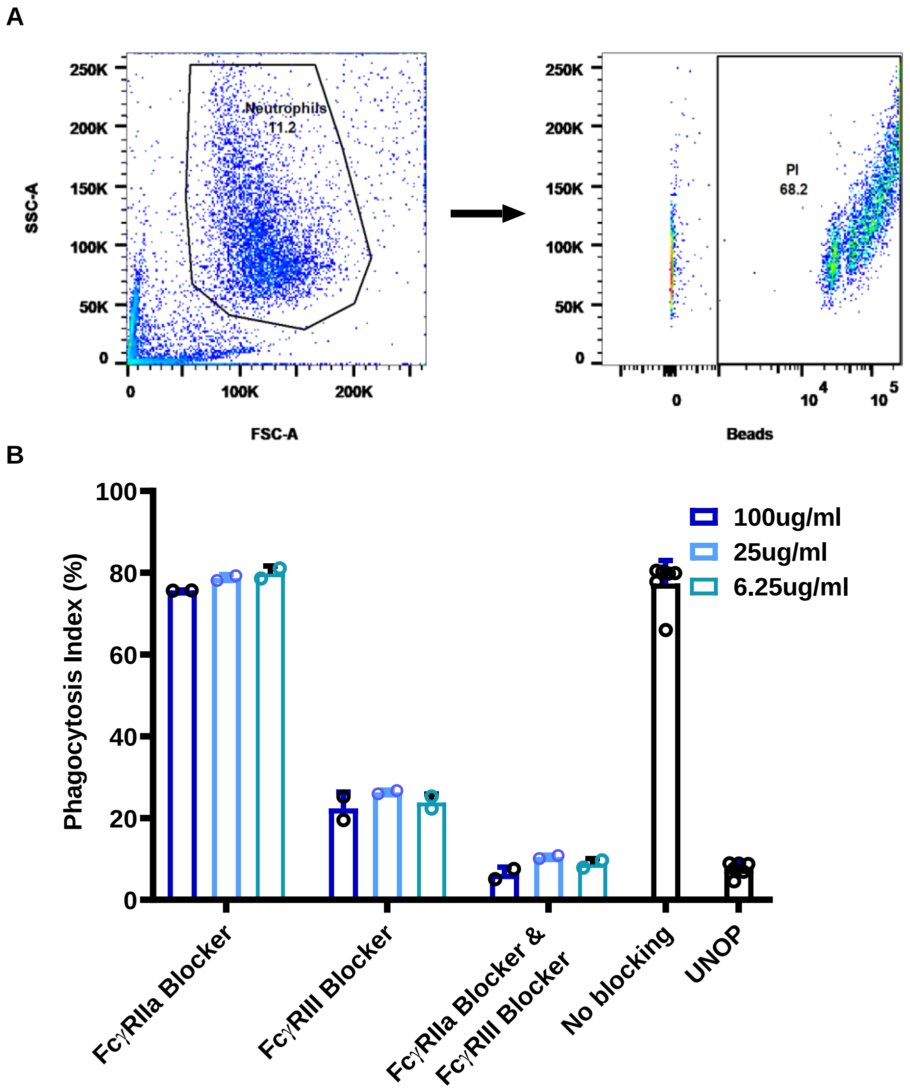

Supplementary Figure 4 Titration of FcγRIIa and FcγRIII blocking antibodies

(A) Example of gating strategy to quantify the level of opsonic phagocytosis by isolated neutrophils. This gating strategy was used in Figures 1E-G, 2A-B, 4A, 4C-E, 5B, 5G, 6 and Supplementary Figures 4B and 6. The neutrophil population was gated on size and complexity (FSC-SSC) and the phagocytosis index (PI) was defined as the percentage of neutrophils that had phagocytosed fluorescent beads.

(B) Neutrophils were blocked by FcγRIIa and FcγRIII blocking antibodies at concentration of 100ul/ml (dark blue bars), 25ug/ml (light blue bars) and 6.25ug/ml (cyan blue bars) prior co-incubation of opsonized CSP-coated beads for phagocytosis. No differences were observed when the FcγRs were blocked by blocking antibodies at different concentrations, suggesting that the lower concentration had reached saturation. Bars and error bars represent the mean and standard deviation of each experiment condition tested in duplicates.

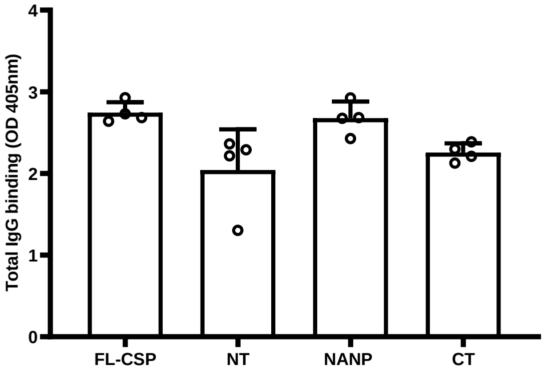

Supplementary Figure 5 Antibody binding to different regions of CSP

Antibodies from Kenyan adult pool were tested for total IgG binding to full-length CSP (FL), the N-terminal region (NT), the NANP repeats (NANP) and the C-terminal region (CT). The antibodies to the N-terminal region showed lower binding compared to antibodies to full-length CSP and the NANP repeats (P=0.015 and P=0.025 respectively, One-way ANOVA), but similar level of antibody binding compared to antibodies to the C-terminal region (P=0.586, One-way ANOVA). Bars and error bars represent the mean and standard deviation of data from 2 experiments repeats conducted in duplicates.

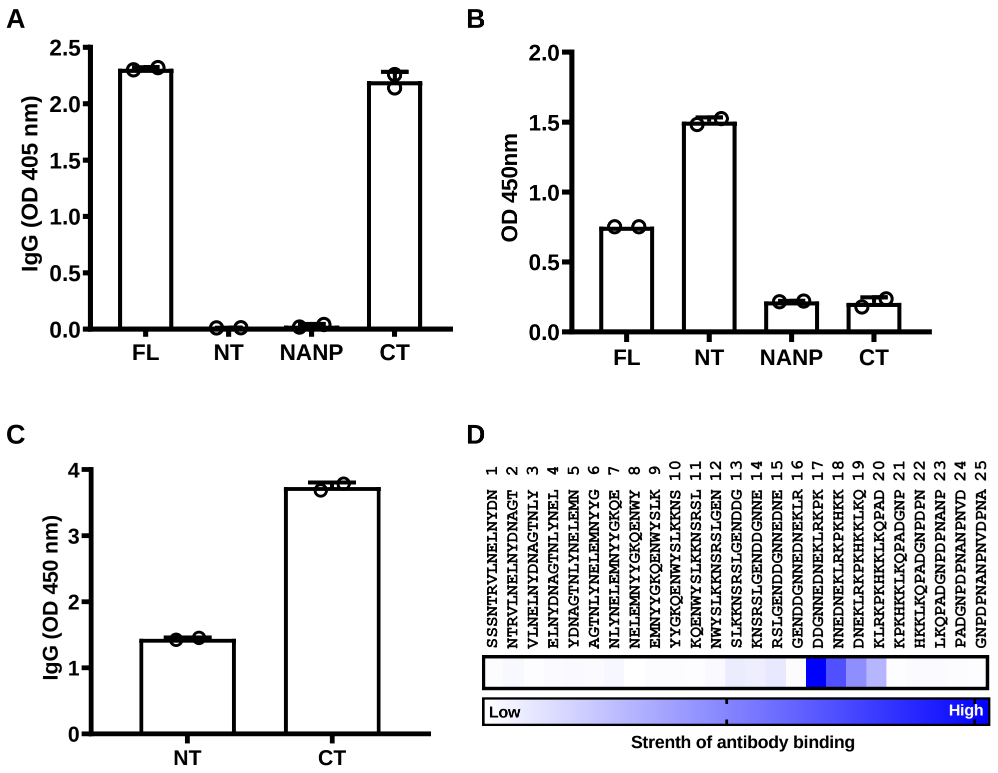

Supplementary Figure 6: Antibodies induced by vaccination of rabbits with different CSP regions

(**A**) Rabbits vaccinated with the N+C region of CSP only generated antibodies to the CT region of CSP, not to the NT region of CSP. Figures show IgG reactivity of antibodies to full length CSP (FL), NT region (NT), central NANP-repeat region (NANP), and CT region (CT), determined by ELISA. (**B**) Rabbits vaccinated with the NT region peptide only generated antibodies to the NT region of CSP. (**C**) The level of IgG reactivity to full length CSP (FL) by rabbit antibodies raised against the NT region and CT region, determined by ELISA. Bars and error bars represent the mean and standard deviation of OD values from two replicates. (**D**) Antibodies from rabbits vaccinated with the N-terminal region of CSP were tested for total IgG reactivity using an overlapping peptide array of the N-terminal region. Significant and strong IgG reactivity was seen to an epitope represented by peptide 17 (P=0.036 for differences in peptide reactivity, Kruskal-Wallis test, 2 experiments). The amino acid sequences for each peptide 1-25 are shown (See also Figure 4F).

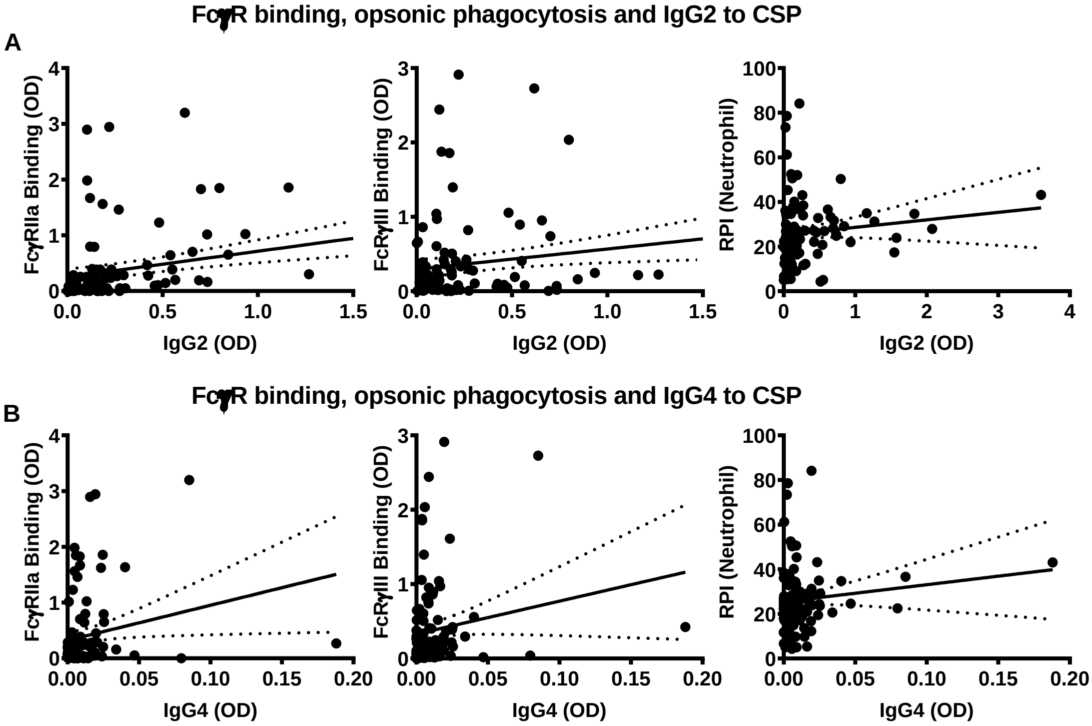

Supplementary Figure 7: Correlation between opsonic phagocytosis, FcγR binding and CSP specific IgG2 and IgG4 subclasses in the Kenyan adult cohort (Kanyawegi cohort, n=104)

(**A**) Correlation between IgG2 to CSP and FcγRIIa, FcγRIII, phagocytosis by neutrophils. Correlation between IgG2 to CSP and FcγRIIa (Spearman's Rho=0.59, P<0.001), FcγRIII (Spearman's Rho=0.30, P=0.002), opsonic phagocytosis (Spearman's Rho=0.26, P=0.007)

(**B**) Correlation between IgG4 to CSP and FcγRIIa, FcγRIII, phagocytosis by neutrophils. Correlations between IgG4 to CSP and FcγRIIa (Spearman's Rho=0.35, P<0.001), FcγRIII (Spearman's Rho=0.32, P<0.001). IgG4 was not correlated with opsonic phagocytosis (Spearman's Rho=0.09, P=0.365)

The solid and dotted lines represent the linear regression line and 95% confidence intervals.

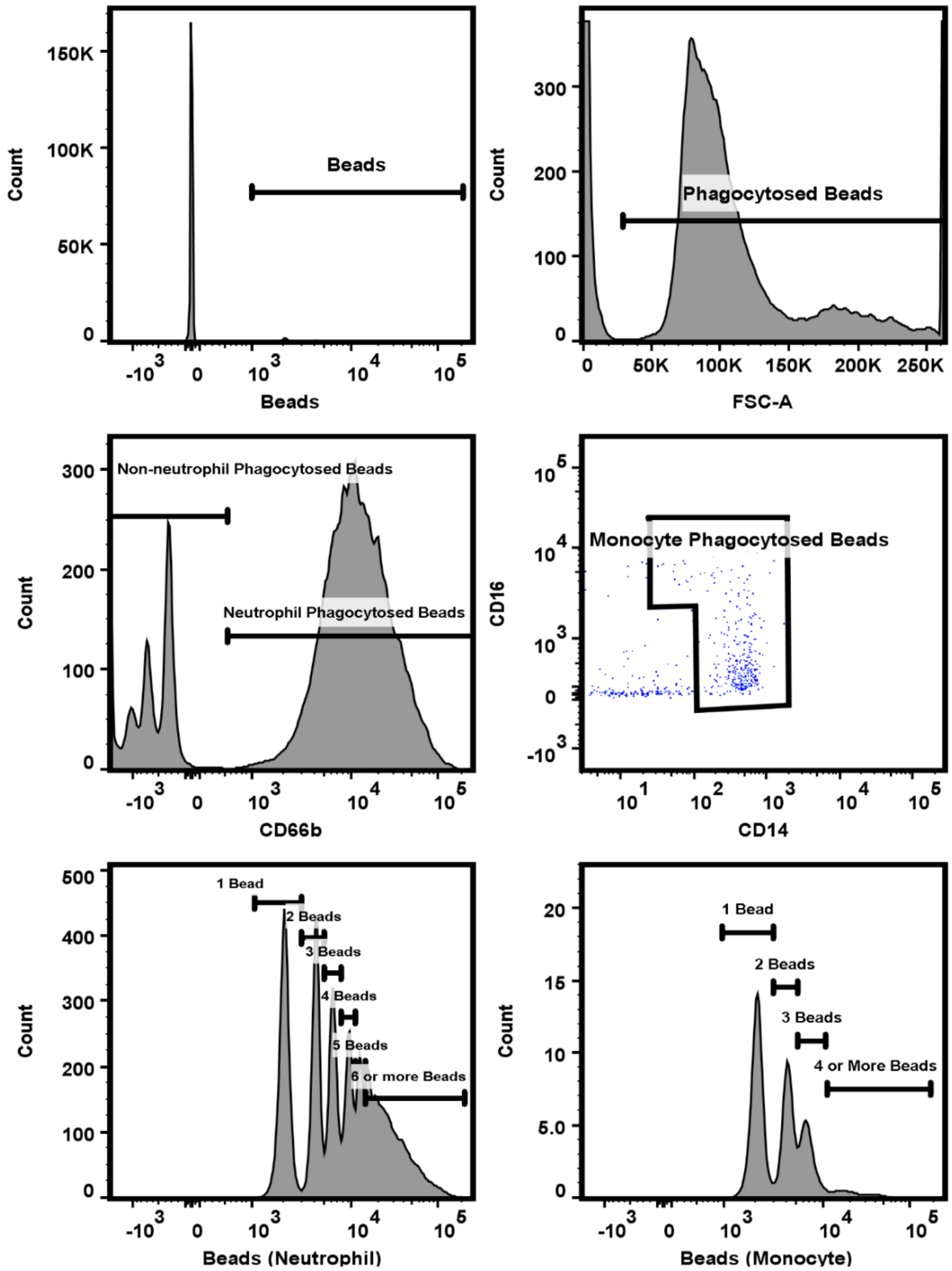

Supplementary Figure 8 Gating strategy for whole leukocyte assay

Gating strategy used to quantify the level of phagocytosis by neutrophils and monocytes (Figures 1A-C). Cells, which phagocytosed beads, were gated based on fluorescent intensity (FITC ^{high}) and size (FCS ^{high}). This population was further divided into neutrophils (CD66b ^{high}) and monocytes (CD66b ^{low} and CD14 $^{+}$). The number of beads phagocytosed by neutrophils or monocytes were determined based on the beads fluorescent intensity. These numbers were further standardized according to the number of phagocytes (including neutrophils and monocytes) acquired in each sample and expressed as beads per 100 phagocytes.

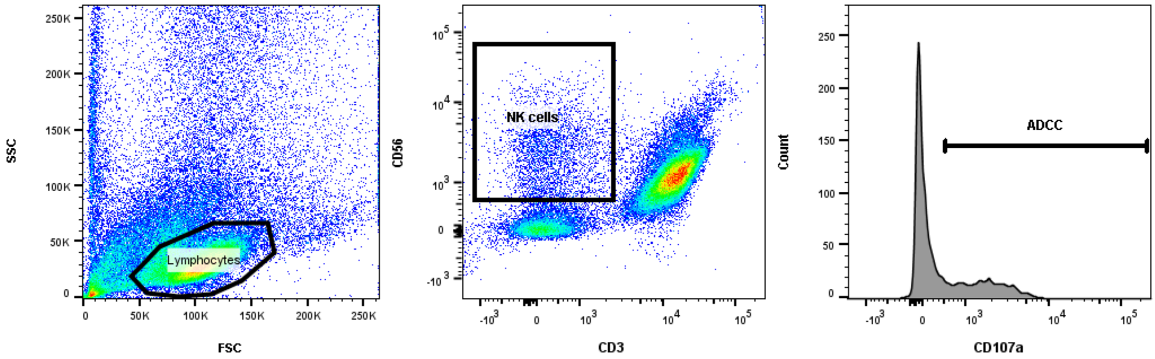

Supplementary Figure 9 Gating strategy for ADCC assay using primary NK cells

Example of the gating strategy used to quantify activation of NK cells (used in Figures 2G-H). The NK cells were defined as $CD3^-$ and $CD56^+$ lymphocytes. The level of NK activation (reflecting ADCC activity) was determined as percentage of CD107a positive NK cells.

Supplementary Table 1 Summary of the Chulaimbo cohort characteristics

Age (year), mean±SD	Children (78.7%, n=59)	2.4±1.8
	Adult (21.3%, n=16)	39.7+18.1
Gender	49.3% Female (n=37)	50.7% Male (n=38)
Parasitemia (Pf/200WBC) Geometric mean (95%CI)	46.2% (n=18) Smear Positive	18.9 (7.1, 49.9)
Hemoglobin (g/dL), mean±SD	Anemia (n=35)	9.5±1.11
	Non-anemia (n=35)	12.6±1.10

Supplementary Table 2: Correlation between total IgG, IgG subclasses to CSP and CSP specific antibody functions in the Kenyan adult cohort

	FcγRIIa Binding		FcγRIII Binding		Phagocytosis by Neutrophils	
	Correlation coefficient	P-value	Correlation coefficient	P-value	Correlation coefficient	P-value
Total IgG	0.69	<0.001	0.75	<0.001	0.50	<0.001
IgG1	0.79	<0.001	0.70	<0.001	0.32	<0.001
IgG2	0.59	<0.001	0.30	0.002	0.26	0.007
IgG3	0.53	<0.001	0.60	<0.001	0.50	<0.001
IgG4	0.35	<0.001	0.32	<0.001	0.09	0.365

Kanyawegi cohort. N=104. Values show Spearman's Rho.

Supplementary Table 3: Multivariate analysis of the associations between opsonic phagocytosis, FcγR binding and IgG subclasses in the Kenyan adult cohort (Kanyawegi cohort)

	IgG1			IgG2			IgG3			IgG4		
	β	95% CI	P-value	β	95% CI	P-value	β	95% CI	P-value	β	95% CI	P-value
FcγRIIa	0.88	0.72, 1.05	<0.001	0.07	-0.08, 0.23	0.353	0.30	0.17, 0.42	<0.001	-0.47	-3.89, 2.95	0.786
FcγRIII	0.65	0.49, 0.81	<0.001	-0.07	-0.21, 0.07	0.339	0.39	0.28, 0.50	<0.001	-1.13	-4..31, 2.05	0.481
Phagocytosis by Neutrophils	4.44	-1.06, 9.94	0.113	-1.38	-6.45, 3.69	0.590	9.77	5.77, 13,76	<0.001	3.94	-109.25, 117.14	0.945

β values: Linear regression coefficient

Kanyawegi cohort. N=104

Supplementary Table 4: Spearman's correlation between opsonic phagocytosis, FcγR binding and IgG subclasses in the Kenyan adults and children cohort (Chulaimbo cohort)

	FcγRIIa Binding		FcγRIII Binding		Phagocytosis by Neutrophils	
	Correlation coefficient	P-value	Correlation coefficient	P-value	Correlation coefficient	P-value
Total IgG	0.39	<0.001	0.28	0.019	0.36	0.002
IgG1	0.31	0.008	0.35	0.003	0.35	0.003
IgG3	0.11	0.37	0.33	0.006	0.33	0.006
FcγRIIa Binding	-	-	-	-	0.22	0.065
FcγRIII Binding	-	-	-	-	0.39	<0.001

Supplementary Table 5: Multivariate analysis of the association between opsonic phagocytosis and FcγR binding Kenyan adults and children cohort (Chulaimbo cohort)

	Phagocytosis by Neutrophils		
	β	95% CI	P-value
FcγRIIa Binding	4.67	-1.79, 11.12	0.154
FcγRIII Binding	47.90	40.50, 55.30	<0.001

B values: Linear regression coefficient

SupplementaryTable 6 Experimental materials and settings of opsonic phagocytosis assays

Targets	Effector cell	Opsonin	Specific experiment condition	Aim of experiment
P.falciparum sporozoites	THP-1 cells	Malaria exposed adult sera	Serum free	Determine whether THP-1 cells can phagocytose antibody opsonized sporozoites
P.falciparum sporozoites	Neutrophils	Malaria exposed adult sera	Neutrophils were used in 2.5% human serum media	Determine whether neutrophils can phagocytose antibodies opsonized sporozoites
P.falciparum sporozoites	Neutrophils	CSP specific rabbit IgG	Neutrophils were used in 2.5% human serum media	Is CSP is a major antibody target for promoting opsonic phagocytosis?
P.berghei sporozoites expressing PfCSP	Neutrophils	Malaria exposed adult sera	Neutrophils were used in 2.5% human serum media	Is CSP is a major target of naturally-acquired antibodies that promote opsonic phagocytosis
P.berghei sporozoites expressing PfCSP or Beads coated with PfCSP	Whole leukocytes	Malaria exposed adult sera or CSP specific rabbit IgG	Leukocytes were used in 2.5% human serum media	Confirming that CSP is a major target of natural acquired antibody to promote opsonic phagocytosis and confirming neutrophils are the key immune cells mediating opsonic phagocytosis of sporozoites
Beads coated with PfCSP or constructs representing PfCSP regions	Neutrophils	Malaria exposed adult sera	Neutrophils were used in 2.5% human serum media	To estimate the level of antibody mediated opsonic phagocytosis in populations
P.falciparum sporozoites or beads coated with CSP	THP-1 cells or neutrophils	Malaria exposed adult sera	THP-1 cells and the neutrophils were treated with different FcγR blockers	To determine the role of each FcγR in opsonic phagocytosis of sporozoites by neutrophils and THP-1 cells
P. falciparum merozoites	Neutrophils	Malaria exposed adult sera	Neutrophils were used in 2.5% human serum media	To determine the acquisition of antibodies that promote phagocytosis of merozoites by neutrophils
