# Supplementary material for: Mechanisms and targets of Fcγ-receptor mediated immunity to malaria sporozoites
Source: Nat Commun. 2021 Mar 19;12:1742. doi: 10.1038/s41467-021-21998-4 (PMC7979888; doi:10.1038/s41467-021-21998-4)
Supplement: Supplementary file 3 — Reporting summary [file 41467_2021_21998_MOESM3_ESM.pdf]

nature research

Corresponding author(s): James G Beeson

Last updated by author(s): Jan 29, 2021

Reporting Summary

Nature Research wishes to improve the reproducibility of the work that we publish. This form provides structure for consistency and transparency in reporting. For further information on Nature Research policies, see Authors & Referees and the Editorial Policy Checklist .

Statistics

For all statistical analyses, confirm that the following items are present in the figure legend, table legend, main text, or Methods section.

n/a	Confirmed	
☐	☒	The exact sample size (n) for each experimental group/condition, given as a discrete number and unit of measurement
☐	☒	A statement on whether measurements were taken from distinct samples or whether the same sample was measured repeatedly
☐	☒	The statistical test(s) used AND whether they are one- or two-sided<br>*Only common tests should be described solely by name; describe more complex techniques in the Methods section.*
☐	☒	A description of all covariates tested
☐	☒	A description of any assumptions or corrections, such as tests of normality and adjustment for multiple comparisons
☐	☒	A full description of the statistical parameters including central tendency (e.g. means) or other basic estimates (e.g. regression coefficient) AND variation (e.g. standard deviation) or associated estimates of uncertainty (e.g. confidence intervals)
☐	☒	For null hypothesis testing, the test statistic (e.g. F, t, r) with confidence intervals, effect sizes, degrees of freedom and P value noted<br>*Give P values as exact values whenever suitable.*
☒	☐	For Bayesian analysis, information on the choice of priors and Markov chain Monte Carlo settings
☒	☐	For hierarchical and complex designs, identification of the appropriate level for tests and full reporting of outcomes
☐	☒	Estimates of effect sizes (e.g. Cohen's d, Pearson's r), indicating how they were calculated

Our web collection on statistics for biologists contains articles on many of the points above.

Software and code

Policy information about availability of computer code

Data collection	No software was used
Data analysis	Statistical analyses were performed using Prism version 7 (GraphPad Software Inc) and STATA version 13.1 (STATACorp). Flowjo software (version 10) was used for analysis of flow cytometry files. This is stated in the manuscript

For manuscripts utilizing custom algorithms or software that are central to the research but not yet described in published literature, software must be made available to editors/reviewers. We strongly encourage code deposition in a community repository (e.g. GitHub). See the Nature Research guidelines for submitting code & software for further information.

Data

Policy information about availability of data

All manuscripts must include a data availability statement. This statement should provide the following information, where applicable:
- Accession codes, unique identifiers, or web links for publicly available datasets
- A list of figures that have associated raw data
- A description of any restrictions on data availability

Data analysed in the preparation of this manuscript and presented in figures and tables is available from the corresponding author upon request. Release of clinical data will require agreement from relevant ethics committees. Plasmodium falciparum sequences were obtained from the public database, PlasmoDB.org

Field-specific reporting

Please select the one below that is the best fit for your research. If you are not sure, read the appropriate sections before making your selection.

Life sciences study design

All studies must disclose on these points even when the disclosure is negative.

Sample size	Experimental studies to test effects of specific antibodies followed standard principles of testing in duplicate, and performing multiple replicate experiments. Statistical testing was conducted to assess significance. Sample sizes for clinical studies had been pre-determined. Prior to sample testing, sample size estimates were performed to ensure there was sufficient power to detect difference between clinical groups (E.g. age), correlations between continuous variables, or differences in the activity of specific antibody types. In other experiments, sample sizes and sample selection were determined to provide representative data for descriptive statistics.
Data exclusions	No data were excluded
Replication	All experimental approaches were assessed for reproducibility and data was tested for reproducibility. This is reported in the manuscript for each figure and data are provided to demonstrate reproducibility and assay error or variance
Randomization	All samples were included for testing. There was no selection or randomization. Multivariate regression was used to control for covariates (See Table 1, S4, S6)
Blinding	During testing, all samples were coded with unique identifiers and investigator was blinded to the clinical data. Data were decoded for analysis. Samples were also tested in a randomised sequence to avoid introducing bias or systematic error.

Reporting for specific materials, systems and methods

We require information from authors about some types of materials, experimental systems and methods used in many studies. Here, indicate whether each material, system or method listed is relevant to your study. If you are not sure if a list item applies to your research, read the appropriate section before selecting a response.

Materials & experimental systems

n/a	Involved in the study	
☐	☒	Antibodies
☐	☒	Eukaryotic cell lines
☒	☐	Palaeontology
☐	☒	Animals and other organisms
☐	☒	Human research participants
☒	☐	Clinical data

Methods

n/a	Involved in the study	
☒	☐	ChIP-seq
☐	☒	Flow cytometry
☒	☐	MRI-based neuroimaging

Antibodies

Antibodies used

Antibody or reagent - Supplier - Catalogue Number - (Validation method)
Goat-anti-human IgG-HRP, Life Technologies 62-8420 (ELISA and WB)
Goat-anti-rabbit IgG-HRP, Millipore AP307P (WB)
Goat-anti-mouse-IgG-HRP, Millipore AP308P (WB and ELISA)
Streptavidin-HRP, Thermo Fisher Scientific, 21130 (WB and IHC)
Mouse anti-human IgG1 HP6069, Life Technologies A10630 (ELISA, IF)
Mouse anti-human IgG2 HP6002, Life Technologies 05-3500 (ELISA, IHC)
Mouse anti-human IgG3 HP6047, Life Technologies 053600 (ELISA)
Mouse anti-human IgG4 HP6025, Life Technologies A10651 (ELISA, IHC)
Anti-CD14-Alexa647 MϕP9, BD Bioscience 560180 (Flow cytometry)
Anti-CD14-APC M5E2, BD Bioscience 555399 (Flow cytometry)
Anti-CD16-BV421 3G8, BD Bioscience 562874 (Flow cytometry)
Anti-CD32-PE 8.26, BD Bioscience 550586 (Flow cytometry)
Anti-CD64-APC-H7 10.1, BD Bioscience 561190 (Flow cytometry)
Anti-CD64 10.1, Merck 217620 (Flow cytometry)
Anti-CD66b-APC-H7 G10F5, BD Bioscience 561645 (Flow cytometry)
Anti-CD3-APC-H7 SK7, BD Bioscience 560176 (Flow cytometry)
Anti-CD56-PE-Cy7 B159, BD Bioscience 557747 (Flow cytometry)
Anti-CD107a-AF647 H4A3, BD Bioscience 562622 (Flow cytometry)

Antibodies generated in-house (at Burnet Institute)
Anti-CD32a IV.3 (Flow Cytometry)
Anti-CD16 3G8 (Flow Cytometry)
mAb2A10-LALA mutant (ELISA, WB)

mAb MGG4 (ELISA)

Antibodies generate in the study (Validation method)
Rabbit-anti-full-length CSP (ELISA, WB)
Rabbit-anti-N+C (ELISA)
Rabbit-anti-NT (ELISA)

Method of validation of reagents is indicated: IF=immunofluorescence, IHC=immunohistochemistry, WB=western blot

Validation	Antibodies were validated using several approaches including use of positive and negative reference controls, immunoblotting, and reviewing available data from commercial suppliers. Antibodies generated in the study were validated by immunblotting and/or ELISA using purified reference antigens. Validation method is indicated for each reagent above.

Eukaryotic cell lines

Policy information about cell lines

Cell line source(s)	Plasmodium falciparum, 3D7 and D10 isolates (obtained from the Walter and Eliza Hall Institute. Validated by PCR and sequencing, and by immunophenotyping). HEK293F cells obtained from commercial supplier (not independently validated). THP-1 cells obtained from ADCC (not independently validated, Fc-gamma-receptor expression was confirmed by Flow cytometry
Authentication	Plasmodium falciparum. Validated by PCR and sequencing, and by immunophenotyping. HEK293F cells were used for expression of recombinant proteins and were not independently validated. Proteins expressed using this cell line were validated by SDS-PAGE, western blotting and or ELISA. THP-1 cells were not independently validated (Fc-gamma-receptor expression was confirmed by Flow cytometry)
Mycoplasma contamination	Plasmodium falciparum, 3D7 isolate, had been previously tested and found to be Mycoplasma free. HEK293F cells were supplied as Mycoplasma free by the supplier, but were not independently tested. THP-1 cells were not tested
Commonly misidentified lines (See ICLAC register)	None used

Animals and other organisms

Policy information about studies involving animals; ARRIVE guidelines recommended for reporting animal research

Laboratory animals	New Zealand White Rabbits (male and female) raised in laboratory facilities were used for vaccination. Use of animals was approved by the Animal ethics committee and care and handling followed relevant guidelines
Wild animals	*Provide details on animals observed in or captured in the field; report species, sex and age where possible. Describe how animals were caught and transported and what happened to captive animals after the study (if killed, explain why and describe method; if released, say where and when) OR state that the study did not involve wild animals.*
Field-collected samples	*For laboratory work with field-collected samples, describe all relevant parameters such as housing, maintenance, temperature, photoperiod and end-of-experiment protocol OR state that the study did not involve samples collected from the field.*
Ethics oversight	Animal ethics committee of the Walter and Eliza Hall Institute, Australia

Note that full information on the approval of the study protocol must also be provided in the manuscript.

Human research participants

Policy information about studies involving human research participants

Population characteristics	Plasma from malaria-exposed healthy, asymptomatic adults living in the Kanyawegi sub-district of Kisumu County, Kenya (n=104, age range 18-79; 26.9% male) was collected in August 2007 (Kanyawegi cohort); no participants had symptoms of malaria and 46.15% had asymptomatic P. falciparum parasitemia (detected by microscopy). Plasma from healthy, asymptomatic young children and adults living in the Chulaimbo sub-district of Kisumu County, Kenya (n=75, 55% female, age range 0.3-5.9 years for children, age range 19.6-69.2 years for adults) was collected in February-March 2007 (Chulaimbo cohort). Transmission intensity was relatively high at the time blood was collected (prevalence of asymptomatic parasitemia in children ≤10 years was 70-80%) Blood was collected for use in leukocyte assays from health adult female and male donors (age range 25-50).
Recruitment	Participants were approached by the study team and invited to participate in the study. Following explanation of the study and formal consent to participate, study enrollment was undertaken. The participants did not self select or self-nominate.
Ethics oversight	Ethics approval was obtained from the Alfred Hospital Human Research and Ethics Committee (protocol 385-18), the Institutional Review Board for Human Investigation at University Hospitals of Cleveland for Case Western Reserve University, USA (protocol 02-04-04) and the Ethical Review Committee at the Kenya Medical Research Institute, Kenya (protocol SSC867).

Written informed consent was obtained from all study participants or their parents or legal guardians.

Note that full information on the approval of the study protocol must also be provided in the manuscript.

Flow Cytometry

Plots

Confirm that:

- [x] The axis labels state the marker and fluorochrome used (e.g. CD4-FITC).
- [x] The axis scales are clearly visible. Include numbers along axes only for bottom left plot of group (a 'group' is an analysis of identical markers).
- [x] All plots are contour plots with outliers or pseudocolor plots.
- [x] A numerical value for number of cells or percentage (with statistics) is provided.

Methodology

Sample preparation

For assays using monocytes and neutrophils, fresh human peripheral blood was collected and treated to deplete red blood cells. No other processing was performed. For assays of NK cells, freshly collected human peripheral blood was used, PBMCs were isoalted and then PBMCs containing NK cells were cultured in RPMI1640 supplemented with 10% FCS and 100 IU/ml of interleukin-2 (IL-2) overnight. Cells were then used in assays.

Instrument

Becton Dickinson FACSVerse and FACS Canto II and LSR Fortessa X20

Software

FlowJo (v 10.4.2) and FACSSuite, and FACSDiva (Becton Dickinson)

Cell population abundance

The source of cells was human peripheral blood. For opsonic phagocytosis assays excamining neutrophils and monocytes, we typically acquired 20-50,000 phagocytes (neutrophils and monocytes), and the relative abundance of cells was 50-60% for neutrophils and 10-15% for monocytes. For ADCC using NK cells we typically acquired 4-6000 NK cells for analysis, with an NK cell abundance of around 5%.

Gating strategy

Monocytes were labelled with anti-CD14-Alexa657 (MϕP9 BD Bioscience) and anti-CD16-BV421 (3G8 BD Bioscience) and neutrophils were labelled with anti-CD66b-APC-H7 (G10F5 BD Bioscience). Gating strategy is shown in Supplementary Figure S2. NK cells were defined as CD3-, CD56+ lymphocytes and the level of ADCC were quantified as percentage of NK cells with CD107a staining by flow cytometry. Gating strategy is shown in (Supplementary Figure S3).

- [x] Tick this box to confirm that a figure exemplifying the gating strategy is provided in the Supplementary Information.
